# Circ_0025908 regulates cell vitality and proliferation via miR-137/HIPK2 axis of rheumatic arthritis

**DOI:** 10.1186/s13018-021-02615-y

**Published:** 2021-07-30

**Authors:** Xiaofeng Wang, Zhiwen Zhang, Haofeng Liang, Ruixiong Chen, Yuliang Huang

**Affiliations:** Department of Traumatic Orthopedics, Institute of Orthopedics, Huizhou Central People’s Hospital, No. 41, North E’ling Road, Huizhou, 516000 Guangdong Province China

**Keywords:** Rheumatic arthritis, circ_0025908, miR-137, HIPK2

## Abstract

**Background:**

Rheumatic arthritis (RA) is an autoimmune disease with bad effects. Recent researches have shown that circular RNAs (circRNAs) could affect the progress of RA, but the mechanism still indistinct. In this work, we explored the roles of circ_0025908 in RA.

**Methods:**

The levels of circ_0025908, microRNA-137 (miR-137), and mRNA of homeodomain-interacting protein kinase 2 (HIPK2) were detected by quantitative real-time reverse transcription-polymerase chain reaction (qRT-PCR) in RA tissues. Meanwhile, the level of HIPK2 was quantified by Western blot analysis. Besides, the cell functions were examined by CCK8 assay, EdU assay, flow cytometry assay, ELISA, and Western blot. Furthermore, the interplay between miR-137 and circ_0025908 or HIPK2 was detected by dual-luciferase reporter assay.

**Results:**

The levels of circ_0025908 and HIPK2 were upregulated, and the miR-137 level was decreased in RA tissues in contrast to that in normal tissues. For functional analysis, circ_0025908 deficiency inhibited cell vitality, cell mitotic cycle, cell proliferation, and immunoreaction in RA cells, whereas promoted cell apoptosis. Moreover, miR-137 was confirmed to repress the progression of RA cells by suppressing HIPK2. In mechanism, circ_0025908 acted as a miR-137 sponge to regulate the level of HIPK2.

**Conclusion:**

Circ_0025908 facilitates the development of RA through increasing HIPK2 expression by regulating miR-137, which also offered an underlying targeted therapy for RA treatment.

## Introduction

Rheumatic arthritis (RA) is a progressive inflammatory autoimmune disease, whose pathogenesis is related to environmental and genetic factors [1]. In developed countries, RA affects women more physically, with an incidence of 1-2%. RA causes severe effects on the body of patients, such as bone erosion, bone destruction, cartilage degeneration, synovial inflammation, and joint stiffness [2–4]. At present, glucocorticoids, non-steroidal anti-inflammatory drugs, and other drugs have great side effects, and the effect is unsatisfactory [5]. Therefore, it is urgent to further study new treatment methods.

Circular RNAs (circRNAs) are a class of structurally stable RNAs with covalent closed circular conformation [6, 7]. Studies have shown that circRNAs exert important effects in the process of many diseases [8, 9]. For instance, circRNA_09505 aggravates inflammation and joint damage in arthritis mice [10]. Besides, hsa_circ_0001859 regulates the progression of human RA [11]. In addition, circ-AFF2 regulates proliferation, inflammatory response, migration, and invasion of RA synovial fibroblasts [12]. However, so far, the specific regulatory mechanism of circ_0025908 on RA is still not clear, which needs further research.

MicroRNAs (miRNAs) are a type of small non-coding RNAs, which could affect the target genes and regulate cellulate processes [6, 7]. According to previous reports, some miRNAs have been reported in human diseases. In addition, microRNAs play a key role in the treatment of tendon injuries [13–15]. Moreover, miR-137 is a therapeutic target for human glioma [16]. Besides, miR-137-3p regulates the progression of prostate cancer [17]. Moreover, miR-137 acts as a tumor suppressor gene in pituitary adenoma [18]. MiR-338-5p regulates the proliferation, apoptosis, and migration of RA [19]. MiR-708-5p promotes the apoptosis of RA [20]. In addition, miRNA-1183 takes part in the pathogenesis of rheumatic heart disease [21]. Nevertheless, our comprehending of the specific effect of miR-137 in RA remains restricted and needs further study.

Homeodomain-interacting protein kinase 2 (HIPK2) is a serine-threonine kinase, which could control many biological processes, such as apoptosis, angiogenesis, and cell proliferation and invasion [22–25]. Previous studies have shown that HIPK2 function is closely related to Alzheimer’s disease, cardiovascular disease, acute myeloid leukemia, and myelodysplastic syndrome [25–27]. However, the underlying mechanisms that affect the relationship between HIPK2 and the behavior of RA cells are still unclear.

In this paper, we discovered the function of circ_0025908 in RA cells. The research reveals that circ_0025908 may facilitate RA development by sponging miR-137 and increasing the HIPK2 levels. In addition, our study may help bridge the gap between basic science and clinical science, thus accelerating the understanding of RA. Translational medicine (TM) is an emerging medical method and a process that effectively promotes medical progress [28, 29]. Our next experiment will verify the role of circ_0025908 in clinical practice in the context of TM, so as to further promote the development of RA targeted therapy research. Our findings could be a new insight into the evolution of targeted therapies for RA.

## Materials and methods

### Clinical samples

The study was audited by Huizhou Central People’s Hospital. Forty pairs of synovial tissue samples from patients with RA and normal tissue were gathered from Huizhou Central People’s Hospital. All patients have written the informed consent. Subsequently, the samples were frozen and preserved at −80 °C for use.

### Cell lines and cell culture

In this study, we isolated cells from normal and RA samples. The tissue is chopped into small pieces and then placed in a tube filled with 2 mg/mL type II collagenase for digestion for 2 h. Whereafter, the cells were collected and were labeled FLS-Normal and FLS-RA.

### Quantitative real-time reverse transcription-polymerase chain reaction (qRT-PCR)

RNAs from 40 paired samples and RA cells were utilized using a Trizol reagent (Sigma-Aldrich, St. Louis, MO, USA). The total RNA was manufactured complementary DNA. Next, the SYBR Green kit (Takara, Tokyo, Japan) was used to calculate qRT-PCR. GAPDH and RNU6 (U6) were employed as controls to normalize circRNA and miRNA expressions. The primer sequences used were as follow: circ_0025908, F: 5′-GTCAGCTAACCACTCGCTCT-3′ and R: 5′-GACTGGACAGGCCTCTCTTT-3′; miR-137, F: 5′-GCCGAGTTATTGCTTAAGAA-3′ and R: 5′-CTCAACTGGTGTCGTGGA-3′; HIPK2, F: 5′-CACAGGCTCAAGATGGCAGA-3′ and R: 5′-GGGATGTTCTTGCTCTGGCT-3′; GAPDH, F: 5′-TCCCATCACCATCTTCCAGG-3′ and R: 5′-GATGACCCTTTTGGCTCCC-3′; U6, F: 5′-CTCGCTTCGGCAGCACATATACT-3′ and R: 5′-ACGCTTCACGAATTTGCGTGTC-3′. Relative expressions were calculated with the 2^−△△Ct^ method.

### Western blot

According to the method described by Hou *et al.*, we carry out Western blot [30]. In line with the instructions, the protein was extracted and observed through the Immuno Star LD (Wako Pure Chemical, Osaka, Japan). The antibodies were listed as follows: anti-HIPK2 (ab108543; 1:2000; Abcam, Cambridge, MA, USA), anti-Ki67 (ab92742; 1:1,000; Abcam), anti-PCNA (ab92552; 1:1,000; Abcam), anti-Bax (ab32503; 1:1,000; Abcam), anti-Bcl-2 (ab32124; 1:500; Abcam), p-NF-_K_B p65 rabbit monoclonal antibody (1:1000; Cell Signaling Technology, USA), anti-Total-NF-_K_B p65 (ab32536; 1:50,000; Abcam), and anti-β-actin (ab8227; 1:1000; Abcam).

### Cell transfection

The si-circ_0025908, the control si-NC, Bio-miR-137, Bio-NC, miR-137 mimics, miR-137 inhibitors, and controls were acquired from Ribobio (Guangzhou, China). HIPK2 overexpression (pcDNA-HIPK2) and control plasmid (pcDNA) were also fabricated by Ribobio. Transfection was implemented through Lipofectamine 2000 (Sigma) in line with the instruction.

### CCK8 assay

For determining cisplatin sensitivity, RA (2.0 × 10^3^/well) cells with diverse transfection were seeded in 96-well plates. In each well, 20 μL of CCK8 (Sigma) was added and incubation was 4 h. The absorbancy with assessing at 450 nm, then, the same method was used to verify the cell viability.

### Cell proliferation assay

In this experiment, 5-ethynyl-29-deoxyuridine (EdU) was enforced to detect the cell proliferation rate. After post-transfection, RA cells were planted into 96-well plates. The EdU proliferation assay was implemented with the EdU Apollo In Vitro Imaging Kit (RiboBio). Then, the cell proliferation rates were detected and analyzed.

### Flow cytometry assay

After post-transfection, RA (1 × 10^6^) cells were cultured in 6-well plates. Annexin V-FITC/Propidium Iodide kit (Sigma) was employed to stain the treated cells. Apoptotic cells were measured using a flow cytometer. Meanwhile, the Propidium Iodide Flow Cytometry Kit (Abcam) was applied to detect the cell cycle of RA cells. According to the protocol, the transfected RA cells were fixed and resuspended. Finally, the stained cells were examined using a flow cytometer. The different DNA content represents the diverse cell cycle phase by the cells.

### Dual-luciferase reporter assay

The targeting sequence between miR-137 and circ_0025908 or HIPK2 was forecasted by Starbase (http://starbase.sysu.edu.cn/). Then, the sequences of wild and mutant circ_0025908 or wild and mutant HIPK2, synthesized from Ribobio (circ_0025908-WT, HIPK2 3′UTR-WT or circ_0025908-MUT, HIPK2 3′UTR-MUT). The luciferase activity was examined.

### RNA immunoprecipitation assay (RIP assay)

RIP assay was applied using a RIP kit (Geneseed, Guangzhou, China) to reveal the relation between miR-137 with circ_0025908 and HIPK2. Briefly, RA cells were incubated with anti-Argonaute2 (anti-Ago2) or anti-IgG for 12 h. Next, the RNA was isolated and qRT-PCR was performed using the RNA as a template to detect the expression level of miR-137, circ_0025908, and HIPK2.

### RNA pull-down

MiR-137 mimic-biotin (Bio-miR-137) and its negative control (Bio-NC) were synthesized from RiboBio. A Pierce Magnetic RNA-Protein Pull-Down Kit (Sigma) was used to identify the relationship between circ_0025908 and miR-137. Briefly, RA cells with diverse transfection were lysed and incubated with the probe-bead complex at 4 °C for 3 h. After, the reaction tubes were placed on the magnetic stand for the collection of the beads. Protein K and DNase A were later used to remove the protein and DNA, respectively. Finally, the RNA was eluted using the RNeasy Mini Kit (Sigma) and qRT-PCR was performed using the enriched RNA as a template to detect the expression level of circ_0025908 and miR-137.

### Enzyme-linked immunosorbent assay (ELISA)

The concentrations of IL-1β, IL-6, and TNF-α produced by RA cells were measured by IL-1β, IL-6, and TNF-α ELISA kits (eBioscience, USA, Cat: 70-EK201B/3-96, Cat: 70-EK206/3-96, and Cat: BMS223HS) in accordance with the manufacturer’s guidelines.

### Immunohistochemistry (IHC) assay

Primarily, the tissues were made into paraffin slides. The slides were then treated for antigen retrieval. Afterwards, tissue slides were quenched and blocked, then incubated with anti-HIPK2 (ab108543; 1:2000; Abcam). Subsequently, specimen slides were mixed with HRP-conjugated secondary antibody (ab205718; 1:1,000; Abcam). Finally, the slides were observed.

### Statistical assay

All data were gathered from at least 3 groups of repeat. Pearson’s correlation analysis was applied to measure the correlation between the two groups. The difference between 2 or multiple groups was examined by the Student’s *t* test or ANOVA by applying SPSS (version 17.0; SPSS Inc., Chicago, IL, USA). *P* value < 0.05 was significant.

## Results

### The expression of circ_0025908 was memorably upregulated in RA tissues and cells

**Fig. 1:**
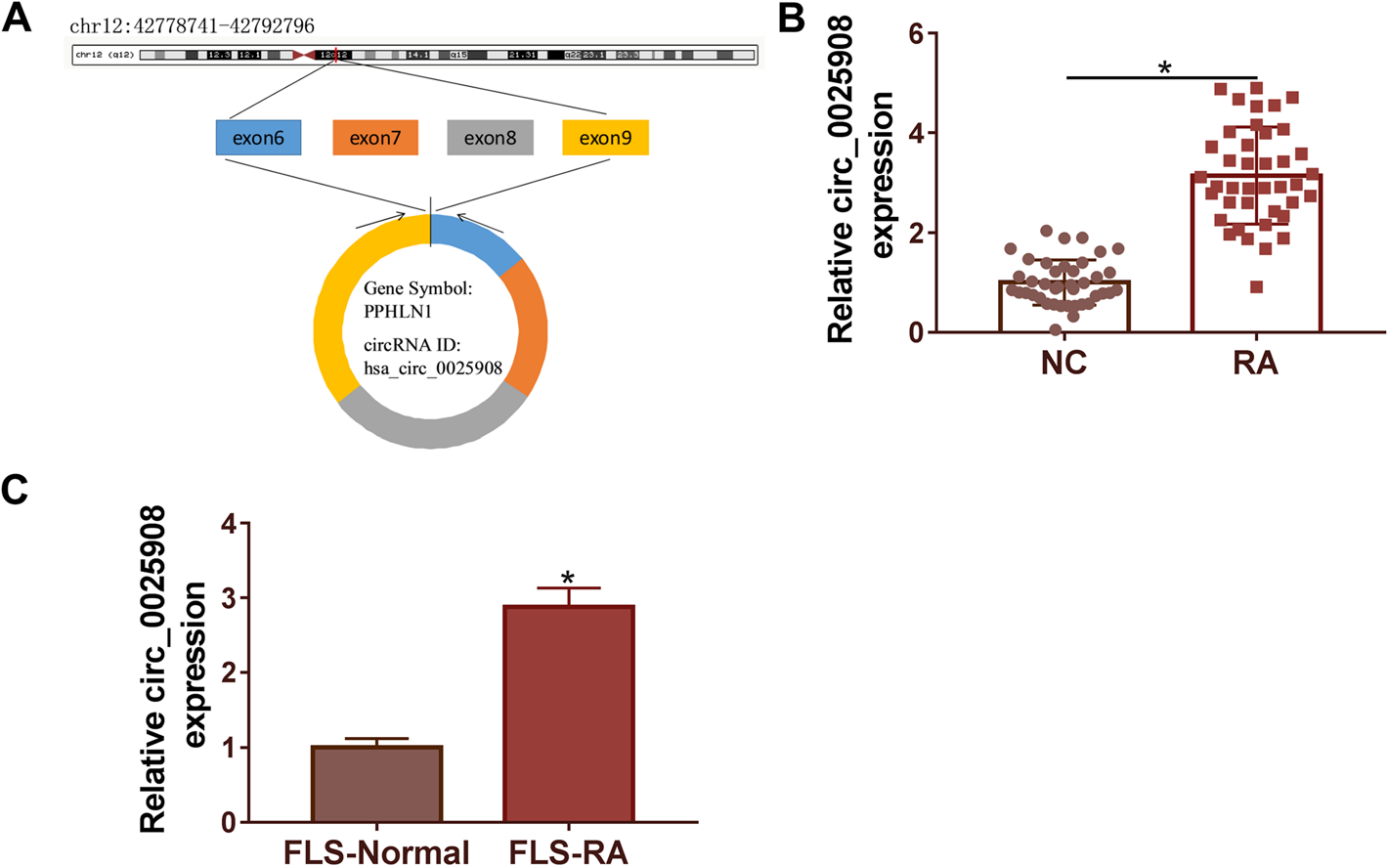
Circ_0025908 expression was enhanced in RA tissues. (**A**) The position of circ_0025908. (**B**) The expression of circ_0025908 in RA tissues (*n* = 40) and normal tissues (*n* = 40) was detected by qRT-PCR. (**C**) The expression of circ_0025908 in FLS-RA cells and control (FLS-Normal) was detected by qRT-PCR. *P < 0.05

As shown in Fig. 1A, circ_0025908 is on chromosome chr12:42778741-42792796. The qRT-PCR assay was conducted to measure the expression level of circ_0025908 in RA tissues (*n* = 40) compared with normal tissues (*n* = 40), and the outcome indicated that the level of circ_0025908 was significantly increased in RA tissues (Fig. 1B). Moreover, our data also suggested that circ_0025908 was upregulated in FLS-RA cell relative to the control cell line FLS-Normal (Fig. 1C). These results revealed the circ_0025908 expression was upregulated in RA tissues and cells, which might take effect in RA.

### Silencing circ_0025908 promoted cell apoptosis, whereas restrained cell vitality, cell mitotic cycle, cell proliferation, and immunoreaction in RA cells

**Fig. 2:**
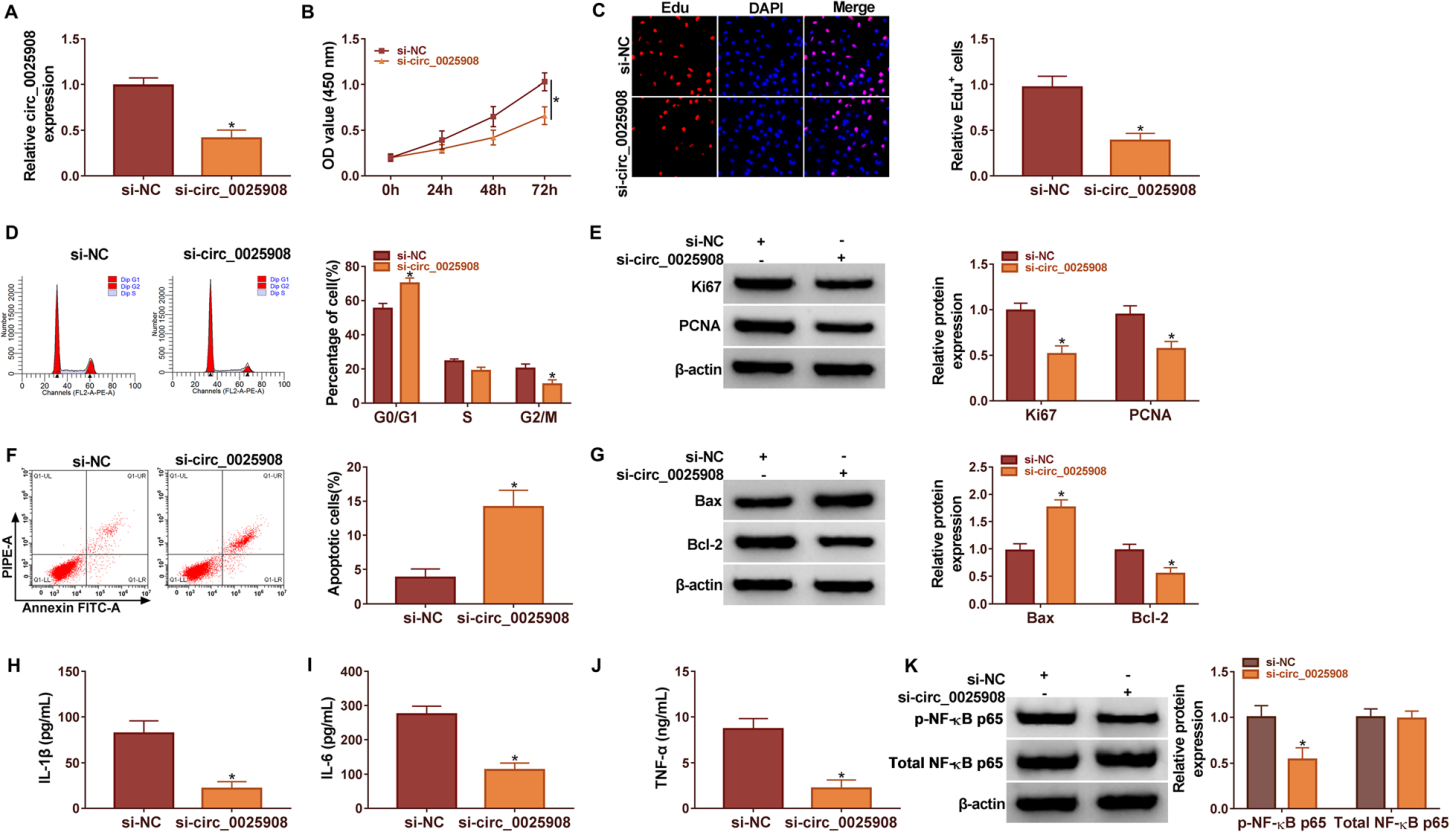
Circ_0025908 knockdown inhibited RA progression. (**A**) The silencing efficiency of circ_0025908 was measured by qRT-PCR. (**B**) CCK8 assay was applied to evaluate the cell viability cells. (**C**) The EdU positive cell was detected by EdU assay. (**D**) Flow cytometry assay was carried out to explain the cell mitotic cycle. (**E**) The protein level of Ki67 and PCNA was examined by western blot. (**F**) Flow cytometry assay was employed to explain the apoptosis of RA cells. (**G**) The protein level of Bax and Bcl-2 were examined by western blot. (**H**-**J**) The level of IL-1β, IL-6, and TNF-α were measured by ELISA. (**K**) The protein levels of p-NF-KB p65 and total-NF-KB p65 were examined by western blot. *P < 0.05

FLS-RA cells were transfected with si-circ_0025908, with si-NC as the control. Transfection efficiency of si-circ_0025908 was detected by qRT-PCR. The result indicated that circ_0025908 expression was memorably restricted in FLS-RA cells transfected with si-circ_0025908 compared to the si-NC group (Fig. 2A). Functionally, the results of the CCK8 assay uncovered the knockdown of circ_0025908 decreased the cell vitality (Fig. 2B). Next, EdU assays unfolded that knockdown of circ_0025908 significantly lessened the cell proliferation of RA cells in contrast to controls (Fig. 2C).

The flow cytometry assay results demonstrated that si-circ_0025908 could significantly block RA cells in the G0/G1 phase (Fig. 2D). Ki67 and PCNA were proved to be involved in cell proliferation. Here, we verified that si-circ_0025908 transfection conspicuously decreased the expression of Ki67 and PCNA (Fig. 2E). Besides, circ_0025908 knockdown remarkably induced cell apoptosis in RA cells (Fig. 2F). Bax and Bcl-2 were proved to be involved in apoptosis of RA cells. We confirmed that si-circ_0025908 transfection conspicuously increased the expression of Bax, but reduced the expression of Bcl-2 in RA cells compared to the si-NC group (Fig. 2G). IL-1β, IL-6, and TNF-α are a class of immune cell cytokines. We indicated that si-circ_0025908 transfection conspicuously decreased the expression of IL-1β, IL-6, and TNF-α (Fig. 2H-J). The expression of p-NF-_K_B p65 and total-NF-_K_B p65 were detected by western blot. The si-circ_0025908 transfection conspicuously decreased the expression of p-NF-_K_B p65, but the expression of total-NF-_K_B p65 barely changed in RA cells compared to the si-NC group (Fig. 2K). Our results indicated that circ_0025908 deficiency might inhibit the cell vitality, cell mitotic cycle, cell proliferation, and immunoreaction in RA cells.

### MiR-137 acted as the target of circ_0025908 in RA cells

**Fig. 3:**
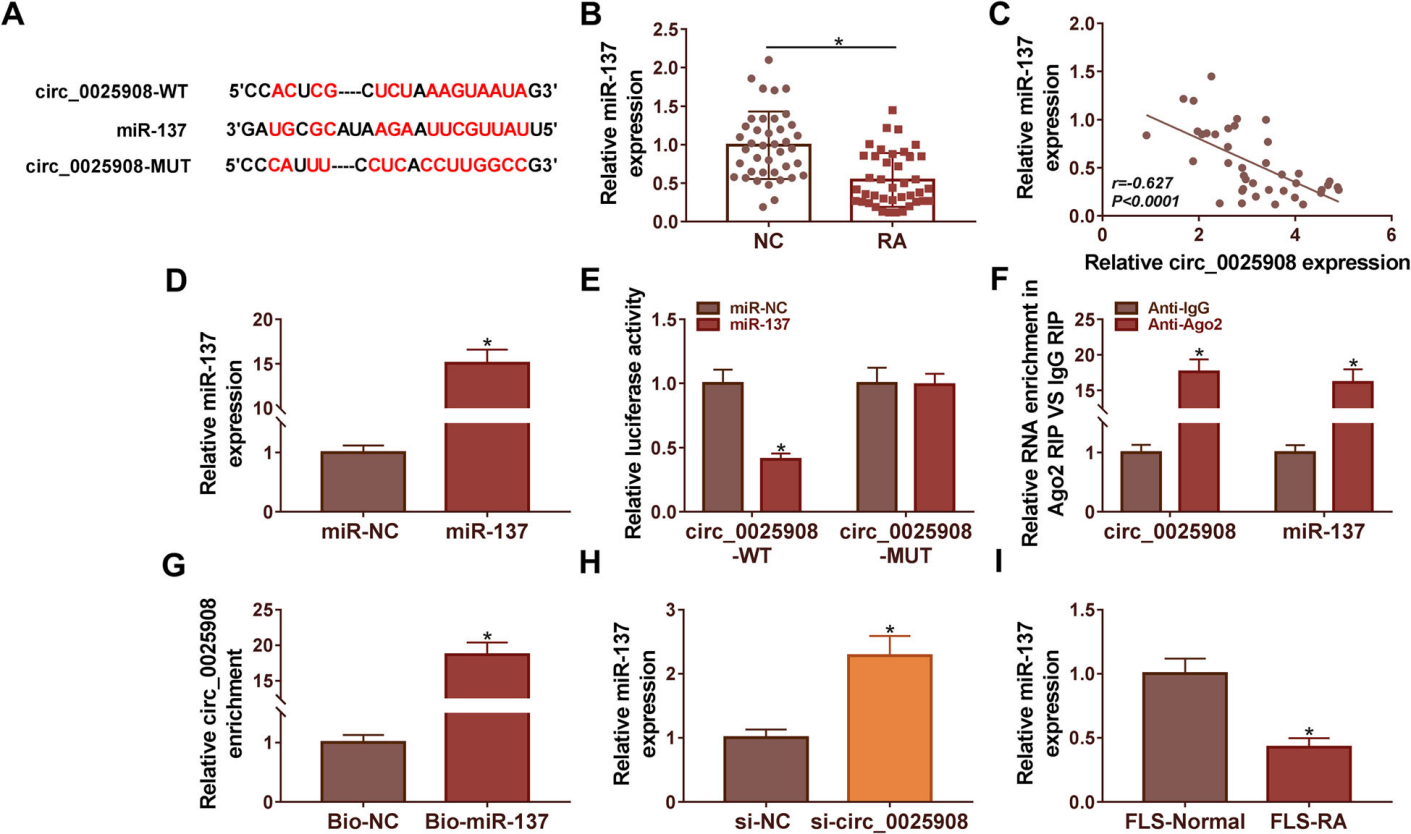
Circ_0025908 acted as a sponge for miR-137. (**A**) The targeted miRNAs of circ_0025908 were forecast by Starbase. (**B**) The expression of miR-137 in RA tissues (*n* = 40) and normal tissues (*n* = 40) was detected by qRT-PCR. (**C**) Pearson’s correlation analysis confirmed that circ_0025908 was negatively associated with miR-137 (*R* = −0.627) in RA tissues. (**D**) QRT-PCR was used to detect the miR-137 expression. (**E**) Dual-luciferase reporter assay was used to verify the relationship between circ_0025908 and miR-137. miR-NC: miR-137 negative control, miR-137: miR-4319 mimic, circ_0025908-WT: circ_0025908 wild type, circ_0025908-MUT: circ_0025908 mutant type. (**F**) RIP assay was used to verify the relationship between circ_0025908 and miR-137. (**G**) RNA-pulldown assay was used to verify the relationship between circ_0025908 and miR-137. (**H**) QRT-PCR was used to detect the miR-137 expression. (**I**) The expression of miR-137 in RA cells was detected by qRT-PCR. *P < 0.05

Starbase was implemented to predict the miR-137 is a target of circ_0025908 (Fig. 3A). The qRT-PCR assay was conducted to measure the expression level of miR-137 in RA tissues (*n* = 40) compared with normal tissues (*n* = 40), the level of miR-137 was significantly decreased in RA tissues (Fig. 3B). Besides, Pearson’s correlation analysis unfolded that miR-137 expression was negatively correlated with circ_0025908 in RA tissues (Fig. 3C). Then, the relative levels of miR-137 in RA cells that were transfected with miR-137 mimics and miR-NC were determined by qRT-PCR. After the addition of miR-137 mimic, the expression of miR-137 significantly increased (Fig. 3D). Results indicated that the luciferase activity was significantly decreased in circ_0025908-WT and miR-137 co-transfected in RA cells compared to miR-NC groups even though no difference was found between circ_0025908-MUT co-transfection groups (Fig. 3E). The RIP assay and RNA pull-down assay further validated the direct reciprocity between miR-137 and circ_0025908 in RA cells (Fig. 3F and G). Moreover, we discovered that the expression of miR-137 was increased by si-circ_0025908 (Fig. 3H). In addition, miR-137 expression was markedly decreased in FLS-RA cells compared with that in FLS-Normal cells (Fig. 3I). In a word, results indicated that circ_0025908 acted as a sponge for miR-137 in RA, and it may play an important role in RA development.

### Circ_0025908 facilitated the progression of RA by sponging miR-137

**Fig. 4:**
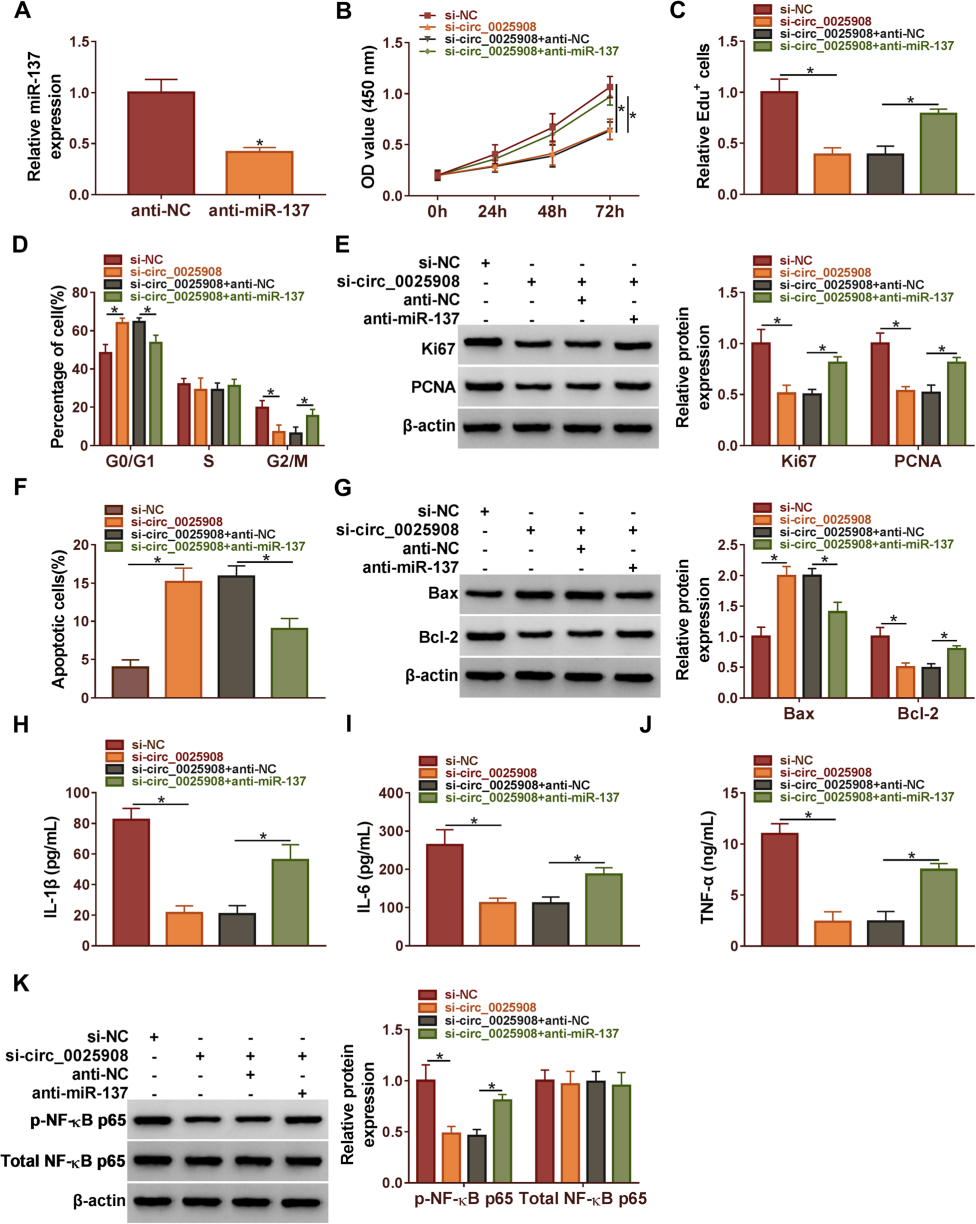
Circ_0025908 facilitated the progression of RA by sponging miR-137. (**A**) The expression of miR-137 was detected by qRT-PCR. (**B**) The cell viability, (**C**) the rate of EdU positive cells, (**D**) the cell mitotic cycle, (**E**) the protein level of Ki67 and PCNA, (**F**) the rate of apoptosis, (**G**) the protein level of Bax and Bcl-2, (**H**-**J**) the level of IL-1β, IL-6, and TNF-α, (**K**) the protein level of p-NF-KB p65 and total-NF-KB p65 were examined by CCK8 assay, EdU assay, flow cytometry assay, ELISA, and western blot, respectively. *P < 0.05

Firstly, qRT-PCR assessed that the expression of miR-137 was significantly decreased by miR-137 inhibitors in RA cells (Fig. 4A). CCK8 assay unfolded that circ_0025908 silencing diminished the cell vitality; however, this effect was dramatically impaired by miR-137 knockdown (Fig. 4B). Meanwhile, cell proliferation was assessed by EdU assays, which manifested that anti-miR-137 restricted the inhibition effects of circ_0025908 silencing in RA cells (Fig. 4C). The flow cytometry assay results demonstrated that si-circ_0025908 could significantly block RA cells in the G0/G1 phase, whereas anti-miR-137 could partially weaken the effect (Fig. 4D). We further implemented a western blot assay, which confirmed that anti-miR-137 restrained the impacts of circ_0025908 silencing on decreased expression of Ki67 and PCNA (Fig. 4E). Besides, circ_0025908 knockdown remarkably induced cell apoptosis, however, this effect was dramatically impaired by miR-137 knockdown (Fig. 4F). We confirmed that si-circ_0025908 transfection conspicuously increased the expression of Bax, but reduced the expression of Bcl-2, whereas the anti-miR-137 restrained the impacts (Fig. 4G). Meanwhile, we indicated that circ_0025908 silencing conspicuously decreased the expression of IL-1β, IL-6, and TNF-α, whereas the anti-miR-137 restrained the impacts (Fig. 4H-J). Moreover, the circ_0025908 silencing notably decreased the expression of p-NF-KB p65, however, the anti-miR-137 lessened the impacts. Meanwhile, the expression of total-NF-KB p65 barely changed (Fig. 4K). In conclusion, our findings demonstrated that circ_0025908 silencing hindered RA development by releasing miR-137.

### MiR-137 targeted HIPK2 in RA cells

**Fig. 5:**
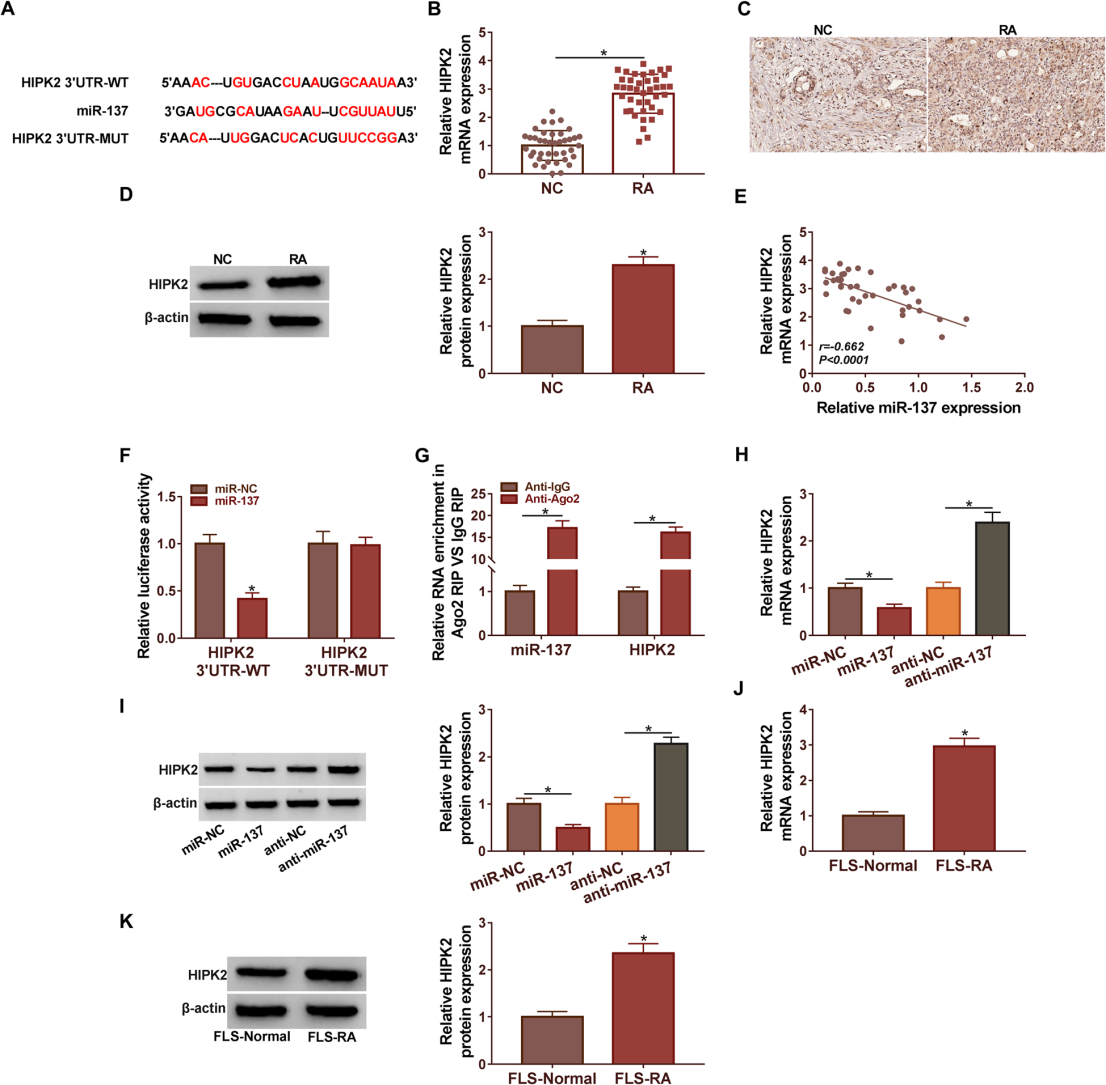
MiR-137 targeted HIPK2 in RA cells. (**A**) The binding site between miR-137 and HIPK2 was analyzed by Starbase. (**B** and **C**) The expression of HIPK2 in RA tissues and normal tissues was detected by qRT-PCR and western blot. (**D**) Pearson’s correlation analysis confirmed that HIPK2 was negatively associated with miR-137 (*R* = −0.662) in RA tissues. (**E**) Dual-luciferase reporter assay was used to confirm the relationship between miR-137 and HIPK2. HIPK2 3′UTR-WT: HIPK2 3′UTR wild type, HIPK2 3′UTR-MUT: HIPK2 3′UTR mutant type. (**F**) RIP assay was used to verify the relationship between HIPK2 and miR-137. (**G** and **H**) The expression of HIPK2 in RA cells was detected by qRT-PCR and western blot. (**I** and **J**) The expression of HIPK2 in RA cells was detected by qRT-PCR and western blot. *P < 0.05

Starbase was applied to predict the binding sites of miR-137 in HIPK2 3′UTR (Fig. 5A). Moreover, the expression level of HIPK2 was examined by qRT-PCR and Western blot in RA tissues. Results showed that HIPK2 expression at mRNA levels and protein levels were remarkably upregulated in RA tissues when compared with control (Fig. 5B and D). Meanwhile, the IHC assay showed the expression of HIPK2 were higher in RA tissues compared with control in specimens (Fig. 5C). Besides, Pearson’s correlation analysis unfolded that miR-137 expression was negatively correlated with HIPK2 in RA tissues (Fig. 5E). Dual-luciferase reporter assay validated that the luciferase activity of HIPK2 3′UTR-WT was notably downregulated after miR-137 mimic transfection. However, the luciferase activity of HIPK2 3′UTR-MUT was not significantly changed by miR-137 (Fig. 5F). RIP assay results demonstrated that firsthand interaction between miR-137 and HIPK2 in RA cells (Fig. 5G). Figure 5H and I showed that compared with the control group, the expression level of HIPK2 is significantly increased by miR-137 inhibitors and significantly decreased by miR-137 mimics. Moreover, we discovered that the expression of HIPK2 was remarkably higher in FLS-RA cells compared with FLS-Normal (Fig. 5J and K). Collectively, these discoveries suggested that miR-137 could interact with HIPK2 to inhibit its expression.

### MiR-137 suppressed RA progression by targeting HIPK2

**Fig. 6:**
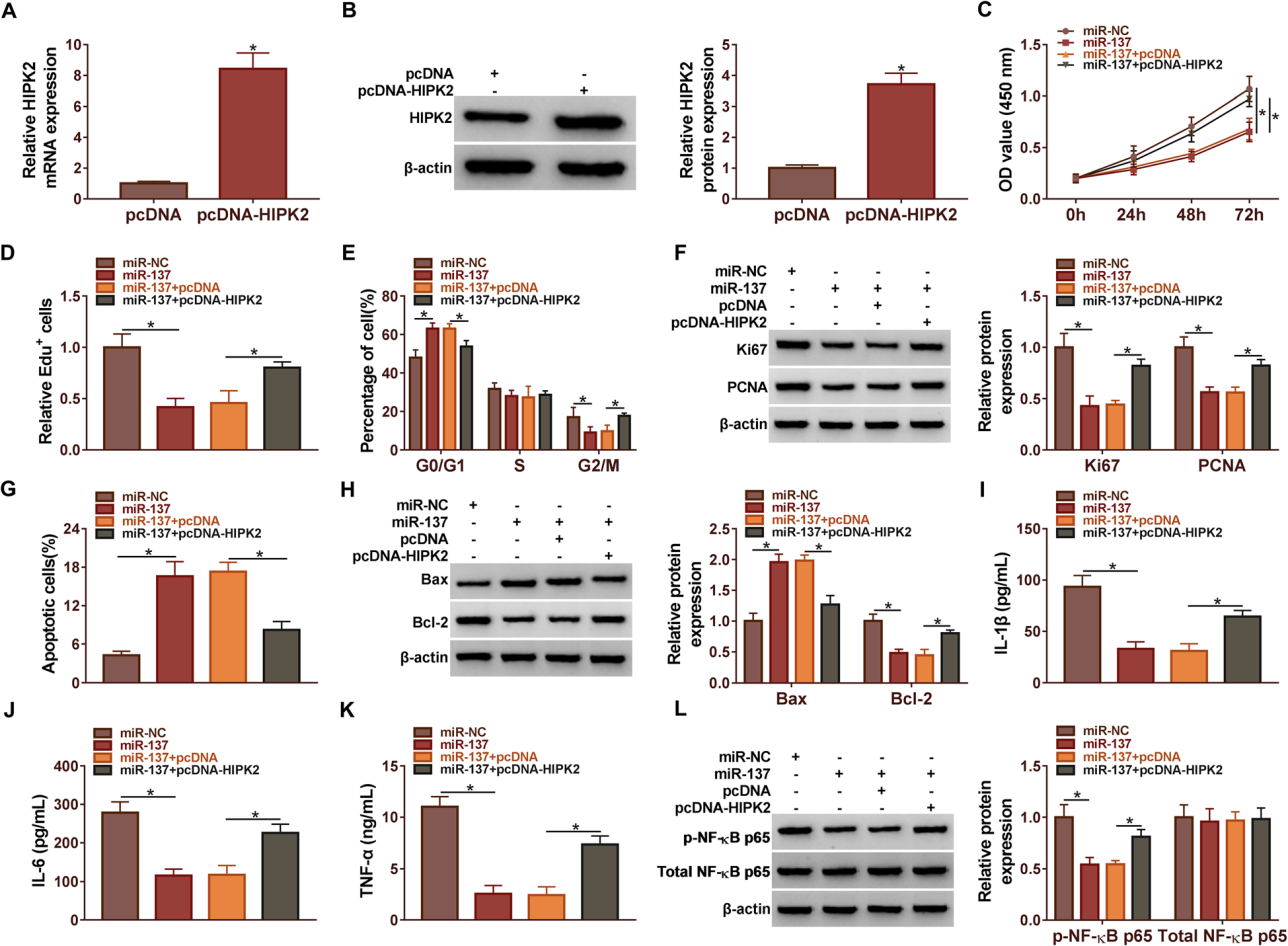
MiR-137 regulated the progression of RA by targeting HIPK2. (**A** and **B**) The level of HIPK2 expression was detected by qRT-PCR and western blot. (**C**) The cell viability, (**D**) the rate of EdU positive cells, (**E**) the cell mitotic cycle, (**F**) the protein level of Ki67 and PCNA, (**G**) the rate of apoptosis, (**H**) the protein level of Bax and Bcl-2, (**I**-**K**) the level of IL-1β, IL-6, and TNF-α, (**L**) the protein level of p-NF-KB p65 and total-NF-KB p65 were examined by CCK8 assay, EdU assay, flow cytometry assay, ELISA, and western blot, respectively. *P < 0.05

Firstly, qRT-PCR and western blot assay assessed that the expression of HIPK2 was significantly increased by transfected pcDNA-HIPK2 compared with the pcDNA group in RA cells (Fig. 6A and B). Subsequently, the CCK8 assay revealed that miR-137 mimic restrained the cell viability, but this impact was significantly attenuated by HIPK2 overexpression (Fig. 6C). EdU assay results confirmed that miR-137 mimic could reduce the cell proliferation of RA cells, an outcome that was antagonistic to the positive effect of HIPK2 overexpression (Fig. 6D). The flow cytometry assay results demonstrated that miR-137 could significantly block RA cells in the G0/G1 phase, whereas HIPK2 overexpression could partially weaken the effect (Fig. 6E). We further implemented western blot assay, which confirmed that HIPK2 overexpression restrained the impacts of miR-137 on decreased expression of Ki67 and PCNA (Fig. 6F). Subsequently, we found that miR-137 mimic facilitated cell apoptosis in RA cells, and this impact was restricted by HIPK2 overexpression (Fig. 6G). Figure 6H showed that the expression of Bax was increased and Bcl-2 was diminished by miR-137 mimic, whereas HIPK2 overexpression could partially lessen these influences. Meanwhile, we indicated that miR-137 mimic conspicuously decreased the expression of IL-1β, IL-6, and TNF-α, whereas the HIPK2 overexpression restrained the impacts (Fig. 6I-K). Moreover, the miR-137 mimic notably decreased the expression of p-NF-KB p65; however, the HIPK2 overexpression lessened the impacts. Meanwhile, the expression of total-NF-KB p65 barely changed (Fig. 6L). In short, all data illustrated that miR-137 regulated the progress of RA cells by targeting HIPK2.

### HIPK2 was regulated by circ_0025908 and miR-137

**Fig. 7:**
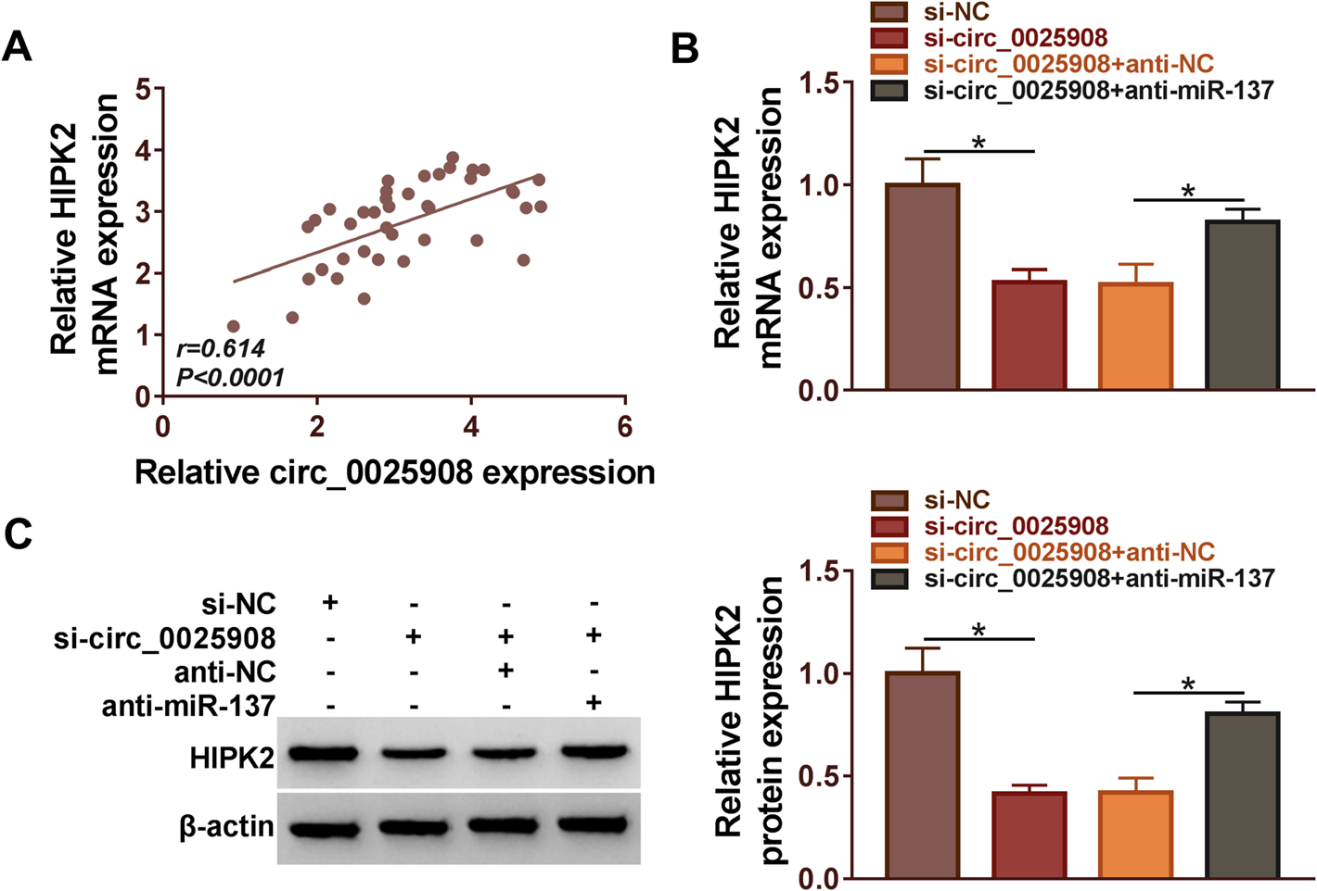
The expression of HIPK2 was regulated by circ_0025908 and miR-137. (**A**) Pearson’s correlation analysis confirmed that HIPK2 was positively associated with circ_0025908 (*R* = 0.614) in RA tissues. (**B** and **C**) The expression of HIPK2 in RA cells was detected by qRT-PCR and western blot. *P < 0.05

Pearson’s correlation analysis validated that circ_0025908 expression was positively correlated with HIPK2 expression (Fig. 7A). Meanwhile, the expression of HIPK2 was diminished by si-circ_0025908, whereas anti-miR-137 could partially lessen these influences (Fig. 7B and C). In conclusion, the expression of HIPK2 was regulated by circ_0025908 and miR-137.

## Discussion

RA is a chronic autoimmune disease that could lead to joint deformity and loss of function and is highly disabling [31]. RA could cause redness, swelling, and pain in the distal joints [32]. Worldwide, the incidence is about 1%, and it is more common in Europe and Asia [33]. RA has become a challenge for people. However, the role of circRNA in RA is still unclear. Therefore, our study investigated the role of circ_0025908.

Previous studies have been discovered that some circRNAs are crucial for RA. For instance, hsa_circ_0001200, hsa_circ_0001566, hsa_circ_0003972, and hsa_circ_0008360 may serve as potential biomarkers for the diagnosis of RA [34]. In our experiment, we determined the circ_0025908 regulates RA progression. Our results indicated that the silencing circ_0025908 induces cell apoptosis, whereas inhibits the progression of RA cells. There have been increasing reports that circRNAs could impact specific gene expression and competitively sponge for miRNAs. For example, circRNA_09505 could target miR-6089 and circFADS2 could regulate miR-498 in RA [10, 35]. In this study, circ_0025908 was observed to accelerate RA progression by sponging miR-137, which is similar to previous findings.

According to previous reports, miR-137 could regulate the progress of glioma, prostate cancer, and pituitary adenoma [16–18]. These results unfold that miR-137 takes part in the development of human diseases. In this paper, we found that miR-137 could regulate the progress of RA. We also manifested the prohibitive role of miR-137 in the development of RA by targeting HIPK2. The results validated that miR-137 may take part in the progress of RA.

The previously reported that HIPK2 plays important role in inflammatory cytokine production [36]. In this work, the expression of HIPK2 was a prominent upregulation in RA tissues and cells. Even more important, we observed that miR-137 upregulation inhibited the development of RA and this impact was lessened by HIPK2. We also unfolded that miR-137 inhibitor repressed the inhibitory effect of circ_0025908 knockdown on HIPK2 level in RA cells. These results are further supporting the regulatory role of the circ_0025908/miR-137/HIPK2 in RA cells.

In a word, the study confirmed that circ_0025908 and HIPK2 were highly expressed and miR-137 was lowly expressed in RA tissues and cells. Furthermore, our study for the first time manifested that circ_0025908 knockdown suppressed RA cell vitality, cell mitotic cycle, cell proliferation, and immunoreaction by regulating the miR-137/HIPK2 axis. This mechanism may be further demonstrated by clinical experiments in the future. The next stage, we will verify the conclusions of this paper in the in vivo joint model and clinical practice, and promote the translation of the research results into clinical practice. We believe that this knowledge could provide a new mechanism for the development of RA treatments.

## Data Availability

Please contact the correspondence author for the data request.
